# Evolution of the Computational Pharmaceutics Approaches in the Modeling and Prediction of Drug Payload in Lipid and Polymeric Nanocarriers

**DOI:** 10.3390/ph14070645

**Published:** 2021-07-05

**Authors:** Shaymaa A. Abd-algaleel, Hend M. Abdel-Bar, Abdelkader A. Metwally, Rania M. Hathout

**Affiliations:** 1 Department of Pharmaceutics, Egyptian Drug Authority, Cairo 12618, Egypt; Shaymaa.abdalgaleel@pharma.asu.edu.eg; 2 Department of Pharmaceutics, Faculty of Pharmacy, University of Sadat City, Sadat 32897, Egypt; hend.abdelbaar@pharma.asu.edu.eg; 3 Department of Pharmaceutics and Industrial Pharmacy, Faculty of Pharmacy, Ain Shams University, Cairo 11566, Egypt; Abdelkader74@yahoo.com; 4 Health Sciences Center, Department of Pharmaceutics, Faculty of Pharmacy, Kuwait University, Safat, Kuwait 13110, Kuwait

**Keywords:** lipid, polymer, simulations, docking, machine learning, in-silico

## Abstract

This review describes different trials to model and predict drug payload in lipid and polymeric nanocarriers. It traces the evolution of the field from the earliest attempts when numerous solubility and Flory-Huggins models were applied, to the emergence of molecular dynamic simulations and docking studies, until the exciting practically successful era of artificial intelligence and machine learning. Going through matching and poorly matching studies with the wet lab-dry lab results, many key aspects were reviewed and addressed in the form of sequential examples that highlighted both cases.

## 1. Introduction

Nowadays, many efforts are exerted in the pharmaceutical field regarding the development of different types of drug delivery systems (DDS). This aims to keep the therapeutic efficacy of the active pharmaceutical ingredient (API), and successfully deliver the proper dose to the physiological target site. Among different types of existing DDS, nanocarriers have gained great interest due to their ultra-small size that controls the pharmacokinetics and pharmacodynamics of the drug [1,2]. Nanocarriers made specifically of lipids and polymers are extensively investigated in the literature, and the number of papers dealing with them is dramatically increasing over time [3,4,5,6]. A core feature for a successful lipid/polymeric formulation is the capacity of the carrier to retain its cargo, i.e., the drug payload [7,8,9]. Selecting the optimum carrier for this purpose is an issue of time, cost and effort if the design is solely built on the basis of wet-lab methods. In silico pharmaceutical formulation design now constitutes a key part of contemporary drug delivery research. It is gaining popularity because it offers the experimentalists a highly pixilated picture of their target based on molecular details of both the drug and the carrier. With this knowledge, designing nanocarriers with optimized properties is much faster with minimal laboratory effort and cost concerns. The scope of the current review is to illustrate the different reported techniques to model and predict he drug payload in the lipid and the polymeric nanocarriers. Evaluation of the consistency of these models with respect to their experimental validation results is another perspective. Given the diversity of APIs and the heterogeneity of lipids and polymeris that are used in nanocarriers preparation, many modelling techniques are described in literature to explain the complex relationship of drug-carrier systems. Some groups emphasized the use of the Flory-Huggins theory and the different solubility models for drug loading prediction. Similarly, increased understanding of the drug-carrier interaction process began reaching its full potential upon reviews discussing molecular dynamics simulations and docking applications in this area. Furthermore, recent related research reports that deal with larger number of parameters tend to apply novel concepts of prediction such as the smart artificial intelligence-based models. Thereby, the aim of this review is to cover all these techniques from naïve primitive approaches to the most innovative ones. The investigated studies include the polymeric and the lipid nanocarriers along with hybrids of them. Each section of the review addresses one modelling area from the perspective of its implications for DL prediction, to the scientific and technical challenges associated with its development. Important literature examples demonstrating these aspects are highlighted. Such a work, appeals to researchers who are interested in the intervention between computer and formulation science to reach state-of-the-art solutions for DL in lipid and polymeric nanocarriers.

**Figure 1:**
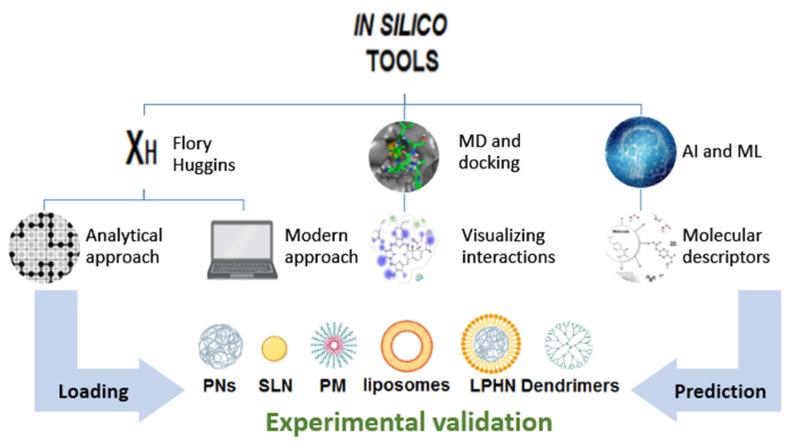
Contribution of different analytical and in silico tools to the drug loading optimization in different classes of nanocarriers.

Along with the review, the reader can find brief definitions and explanations of the mentioned tools. Furthermore, some hints are provided that may help future tuning of technique selection. Figure 1 depicts the scope of this review.

## 2. Solubility Parameters and Flory-Huggins Theory

The essence of DL optimization is to predict molecules’ preferences in excipients depending on their physicochemical properties [10,11]. These are empirical mathematical equations parameterized to wrap the physical details and some structural integration of the involved molecules [11]. The most frequently noticed one is the Flory-Huggins theory (FH). It is a thermodynamic model first introduced in the world of polymers to explain their behavior in solutions [12], and this is well documented in polymers literature [13,14,15]. Recently, this technique was used to extract valuable information regarding the plausible loading of many drugs in polymeric [16,17,18,19] and even lipid [20]—carriers via predicting their mutual solubility/miscibility. It is well known that one of the most important factors that determine the loading capacity of a carrier is the solubility of the drug in this carrier [10,21,22]. Thus, this model depends on estimating specific solubility parameters (SP), usually Hildebrand and Hansen SP, to calculate the final FH interaction parameter (X_H_) as follows:

(1) $$X_{H}=(\delta \;\mathrm{polymer}/\mathrm{carrier}-\delta \;\mathrm{drug}{)}^{2}\times Vd/RT$$

where δ is the solubility parameter, Vd is the molar volume of the drug, R is the gas constant and T is the temperature in kelvin [12].

Assuming constant T and R, the equation clearly stated that the difference in solubility parameters between the drug and the carrier (Δδ) constitutes the driving force of the model. In other words, components with nearly the same solubility parameters are potential companions and vice versa. Actually, the threshold of Δδ below which components are considered soluble slightly differs among published papers. For uniformity, a difference of fewer than 4 MPa^1/2^ was commonly considered as the best approximation that represents all the studied cases. Because of the extensive investigation of this old model, examples are limited to the most recent applications and do not cover all similar cases (Table 1).

### 2.1. Classical Approach

#### 2.1.1. Analytical Procedure

Both solubility parameters (Hansen and Hildebrand) have been used in FH models as they are closely related [23,24]. It is worth mentioning that the group contribution method (GCM) reported by Fedros [25] and Hoftyzer/Van Krevelen [12] is the most common method used for the analytical estimation of SP. It is based on determining the cohesive energy density (CED) in the form of polar, dispersion and hydrogen bond (HB) contributions of various chemical groups -so the name of the method- within a molecule. As shown in Table 1, the first three examples used GCM-based SP along with X_H_ to predict several drugs payload in different nano-formulations using different carriers. In Ghitman et al. work [17], reasonable exponential correlation coefficients between entrapment efficiency (%EE) and X_H_ were obtained with better outcome in case of LPHN (R^2^ of 0.86) c.f. PLGA nanoparticles (R^2^ of 0.61). In contrast, Δδ values obtained by Sun et al. [18] did not distinguish between high and low DL in the PLA core of the prepared PEG-PLA micells. Similarly, their X_H_ values did not provide a well-defined miscibility region and therefore could not predict well the DL capability of these micelles (Table 1). But for Raveendran et al. [19], values of Δδ and X_H_ served well in predicting the polymer core with the highest curcumin payload among four synthesized polymers (Table 1).

#### 2.1.2. Software

Currently, different computer software packages are accommodating computational modules for easier, more interactive and more user-friendly estimation of these contributions. Examples include Hansen Solubility Parameters in Practice (HSPiP) package, Molecular Modeling Pro and Maple software. It is important to mention that using these programs do not contribute in enhancing the performance of the model itself, and the same limitations -like handling very polar and large molecules- still exist. For example, Makoni et al. [20] used HSPiP software to calculate Hansen SP of different drugs and lipid carriers prior to optimization of SLN. Some values were consistent with the experimental results (like Geleol for efavirenz), while others were inconsistent (Table 1). Authors concluded that simple SP predictions could not be used alone to identify the lipid with the best solubilizing potential for a specific drug.

### 2.2. Computational (Modern) Approach

To this end, the classical representation of these solubility models needs upgrading to more explicit versions by incorporating precise modeling techniques. Molecular dynamics simulations (MD) and molecular docking calculations could enhance the performance of solubility models towards complex systems [26]. That is because, MD treats both entities of the model (carrier and drug) in a dynamic manner over time, which means calculating the fine movements of atoms in addition to the rotation, stretching and bending of the flexible bonds [27,28]. Also, instead of representing the carrier in the form of a single unit, MD simulations allow repeating this unit (many chains of building units) to form a 3D structure of interacting, energy minimized carrier molecules that resemble the actual case in the prepared nanoparticle. This tuning of volume difference along with mobility considerations allows catching interactions equally between neighbors. Now, docking is able to provide the binding energy (∆G) between the drug and its carrier based on the whole space and geometry of the carrier, all possible orientations of the drug inside the carrier, and of course accurate physical interactions.

On this basis, an early comparative study was designed by Patel et al. [29] using two approaches for calculating the FH interaction parameter between two drugs and a series of different molecular weights of PEO-PCL copolymer. The first approach was the traditional GCM, and the second was MD simulation. Using Materials Studio (MS) software, MD simulation was applied to the polymer and both API in their pure component form. The output trajectory files were used for extracting CED for all structures and subsequent calculation of FH interaction parameter according to Equation (1). Compared to the analytical GCM which was reported to give paradoxical results to the observed solubility of fenofibrate and nimodipine in liquid caprolactone, MD simulation results were consistent with the experimental validation (Table 2). These findings confirmed the superiority of modern approaches in the correct prediction of solubility and FH interaction parameters.

**Figure 2:**
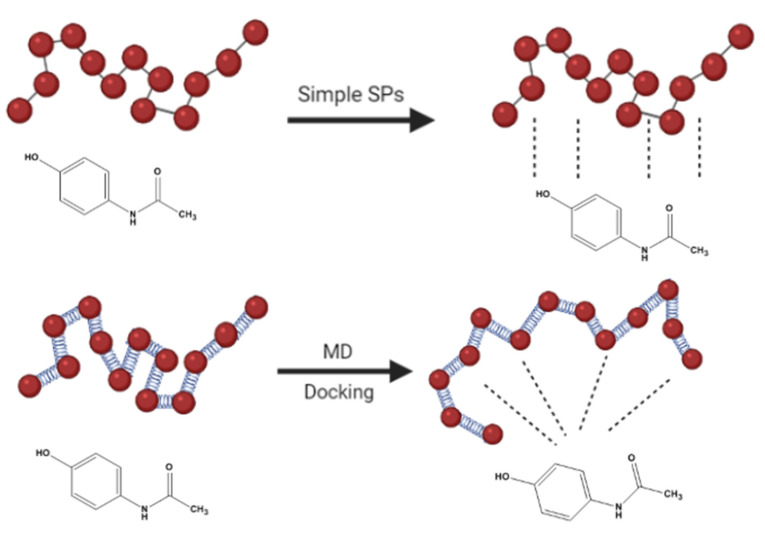
Schematic presentation to contrast classical drug-carrier interaction (solubility parameters) with the modern 3D representation (molecular dynamics and molecular docking).

By the exact same computational protocol, Meunire et al. [30] predicted the loading of six anticancer drugs inside the PLA core of PLA-PEG nanoparticles using the FH interaction parameter. A relatively reasonable exponential correlation between theoretical and experimental data was obtained despite being not completely satisfying for the author (Table 2). The reason was that the author noticed more dispersion in the correlation at low loading hydrophilic compounds especially SAR molecule (outlier) which scored higher than expected X_H_ value. (Table 2). He suggested alternative techniques implementing more relevant algorithms that consider the potential energy of the entire drug-carrier complex besides its pure components such as the mixing energy (E_mix_), for better results. Figure 2 demonstrates in a schematic way the contrast between the classical drug-carrier interaction (solubility parameters) with the modern 3D representation (molecular dynamics and molecular docking).

In testing this hypothesis, it was noticed that Machacˇkova et al. [31] used this modelling algorithm (E_mix_) to evaluate cyclosporine-A miscibility with six virtually built polymers. Using *Blends* module in MS software, a docking method based on the generalization of the FH theory was applied. The energy obtained after calculations is so called the mixing energy (E_mix_) and leads to X_H_ by replacing the numerator of Equation (1) to be E_mix_. The values of X_H_ revealed that both cellulose and chitosan seem to be the best carriers for the modeled drug c.f. other polymers (Table 2). These results were in accordance with previous experimental reports cited by the author, but it is always recommended to consider differences in laboratory protocols while citing experimental validation. The experimental tests remain an essential tool that is usually used to validate the simulation results (should not be thought of as an alternative to experiments), but the two approaches should be seen as complementary.

## 3. MD Simulations and Docking

Simulations and docking obviously add merits of power and accuracy to the prediction models. Recently, these techniques have come into play and show up in published papers as shifting paradigms in modelling nanocarriers. This new concept significantly aids the pre-formulation stage utilizing knowledge-based procedures and hence reaching better formulation targets.

### 3.1. Screening

Many reports confirmed the profound role of MD simulations and docking in the pre-formulation screening of carriers and in identifying candidate drugs expected to have high loading capacity towards specific carrier. This approach makes the whole formulation process extremely time and cost-effective with accurate prediction results that compare fairly well with experimental data.

#### 3.1.1. Carriers-Oriented Screening

For instance, Yadav et al. [32] screened curcumin against five polymers using MD sim ulation and docking. Observations indicated that chitosan outperformed other tested polymers with an excellent ∆G pattern (Table 3). Chitosan was therefore selected for the experimental design of that study. Such a finding was not surprising as Dhanasekaran et al. [33] previously experienced the same results. Curcumin docking results showed a lower ∆G in chitosan c.f. chitin (Table 3). This was in good agreement with the experimental findings, which interestingly offered the maximum capacity of a carrier that can be experienced with any drug, i.e., about 100%.

Similarly, Aparna et al. [34] have picked gelatin as a polymeric carrier for the delivery of amphotericin B based on an in silico outcome. Among eight different evaluated amino-functionalized polymers, gelatin showed the highest docking score for amphotericin B (Table 3). The experimental EE and loading of amphotericin B in gelatin nanoparticles were found to be highly accepted.

Ahmed et al. [35] also screened fluticasone against different polymers prior to the production of stable fluticasone nanoparticles. MD simulation and docking were performed. PVP stabilized HPMC and PVP stabilized Eudragit complexes scored the best ∆G results (−35.22 kcal/mol and −25.17 kcal/mol respectively) c.f. other simulated polymeric structures. After preparation of these nanoparticles, EE percentage was >90% confirming the in silico optimization.

Furthermore, a certain E-coli endotoxin structure was in silico screened by Altintas et al. [36] against twenty-one monomer building blocks commonly used for the preparation of molecularly imprinted polymeric nanoparticles (nanoMIPs). According to the results, itaconic acid, methacrylic acid and acrylamide with ∆G values of −52.24 kcal/mol, −41.43 kcal/mol and −39.87 kcal/mol, respectively, were selected. Experimentally, surface plasmon resonance (SPR) signaling revealed an affinity pattern of 99.5 RU, 66.4 RU and 35.6 RU for itaconic acid, methacrylic acid and acrylamide respectively. The experimental analysis of nanoMIPs for endotoxin detection by SPR sensor yielded results of similar pattern and conforming well to the computational modelling experiments (Table 3).

#### 3.1.2. Drugs-Oriented Screening

A combination of MD simulations and docking calculations was also used to test the stable incorporation of curcumin, paclitaxel and vitamin D3 in PEG-Tyrosine derived polyarylates-PEG carrier. [37]. For the three drugs used, ΔG results that were computationally obtained, were correlated with the maximum drug loading as measured experimentally (Table 3), and a strong linear correlation (R^2^ = 0.93) was observed.

In other similar paper works, Gayathri et al. [38] and Geetha et al. [39] investigated the loading of three antibacterial drugs and three anticancer drugs, respectively, inside chitin nanocarriers. The antibacterial drugs and anticancer drugs were subjected to docking using the same modeled chitin nanocarriers. In both papers, the DL values of the three antibacterial drugs (8.9%, 5.6% and 3.5%) and the three anticancer drugs (3%, 2% and 1%) followed a similar trend with respect to ΔG data (−8.1, −7.3, −5.1 and −6.9, −6.5, −5.4 kcal/mol respectively). Excellent correlations between computational and experimental data were obtained with R^2^ of 0.85 for the antibacterial drugs and 0.93 for the anticancer drugs.

In the same context, Nosrat M. Mahani [40] tested the ability of PLGA to accommodate nine potent, but unstable, thiazoline derivatives with different alkyl and aryl substitutions. Using the hybrid ONIOM calculation reported before [41], all derivatives exhibited negative binding energies indicating good interaction with PLGA. Substitution with R = CH3 and Ar = 1-Methyl-1H-tetrazol-5-yl or 1-Phenyl-1H-tetrazol-5-yl in derivatives 3 and 7 respectively caused more potent binding interactions between the drug and the polymer (best ΔG results) (Table 3). This led to the final selection of these moieties for the successful preparation of stable, biologically active antimicrobial/anticancer formulations.

Talking about stability, Tais et al. applied MD simulations and docking studies to predict the most stable stoichiometric inclusion complex of naringenin with cyclodextrin (CD) [42]. Their main goal was to enhance the solubility of this poorly water soluble drug -and hence its dissolution rate- by high encapsulation inside CD with minimum dissociation rate. Three stoichiometric ratios of naringenin to CD (1:1, 1:2 and 2:1) were then in silico tested. Naringenin was docked on the energy minimized (MM+ FF) CD structure using Polak-Ribiere algorithm in HyperChem^®^ software. Results showed that the 1:1 stoichiometry produced the most stable complex with stabilization energy of 150 and 165 kcal/mol (for hydroxyphenyl ring and chromanone ring orientations of naringenin respectively) c.f. 280 kcal/mol for the 1:2 stoichiometry. The 2:1 stoichiometry was not stabilized due to steric hinderance. Further MD simulations of the most stable complex, i.e., 1:1 stoichiometry, demonstrated conformation change of the chromanone ring orientation to the more stable hydroxyphenyl ring orientation with little or no mobility of the hydroxyphenyl ring orientation at the end of the simulation run. These data confirm the docking results obtained above.

With the same modeling package regarding structure optimization, docking algorithm and MD simulations mentioned previously, Thaiene et al. predicted the stability of Aluminium-chloride-phathalocyanine (AlCIPc) inclusion complex with βCD and its derivative hydroxypropyl-βCD [43]. In general, the 1:1 stoichiometry produced more stable complexes in both AlCIPc-βCD and AlCIPc-hydroxypropyl βCD c.f. stoichiometries 1:2 and 2:1. More specifically, there was no significant difference between stabilization energies of both complexes in 1:1 stoichiometric ratio especially in aqueous medium (−1064 to −1047 kcal/mol and −959 to −944 kcal/mol). Hydroxypropyl βCD was selected for the experimental study.

The ability of a closely related polymer to PLGA, PLA, to interact with another group of drugs was demonstrated in the aforementioned report by Meunire et al. [30]. Being unsatisfied with the results of SP/FH modelling utilizing the solubility parameters described earlier (Table 2), the authors remodeled the same six investigated API using a full Monte Carlo algorithm. This time, they found a strong linear correlation (R^2^ = 0.82) between the obtained ΔG data and the experimental drug loading (Table 3).

Another example of multidrug modelling was introduced by Hathout et al. [44] using gelatin as a principle matrix. Ten API were docked on the gelatin matrix simulated by MD, and the obtained ΔG data were correlated with the experimental masses of the loaded drugs. This model was highly fitting associated with an R^2^ approaching 1 (Table 3).

On a larger dataset comprising a lipid carrier, Metwally and Hathout modelled the loading of twenty-one API in tripalmitin and PLGA 50:50 based nanocarriers simulated by MD [45]. Ten drugs were assigned for the PLGA system and eleven for tripalmitin. Obvious correlations between docking ΔG data of the tripalmitin and PLGA loaded drugs and their corresponding reported drug loading were obtained (Table 3). Furthermore, the authors validated the predictability of the constructed models using a model drug, curcumin. The calculated percentage bias between the actual and the predicted loading values were 12% and 2.03% for tripalmitin SLN and PLGA nanoparticles, respectively.

A similar study was reported by the same authors using the same group of drugs in the tripalmitin based system but with a different docking protocol [46]. The new protocol applied ASE scoring function implemented in Molecular Operating Environment (MOE) software. This score is quietly different from the Ascore implemented in ArgusLab as it considers the distances between the ligand atom-carrier atom pairs in the form of a Gaussian function [46]. Accordingly, a comparable R^2^ for the constructed ΔG-loading correlation (0.86) was found with lower bias percentage (7.71%). This data furnished evidence for the effect of different docking protocols (scoring functions equations) on the accuracy of modelling nanocarriers.

**Figure 3:**
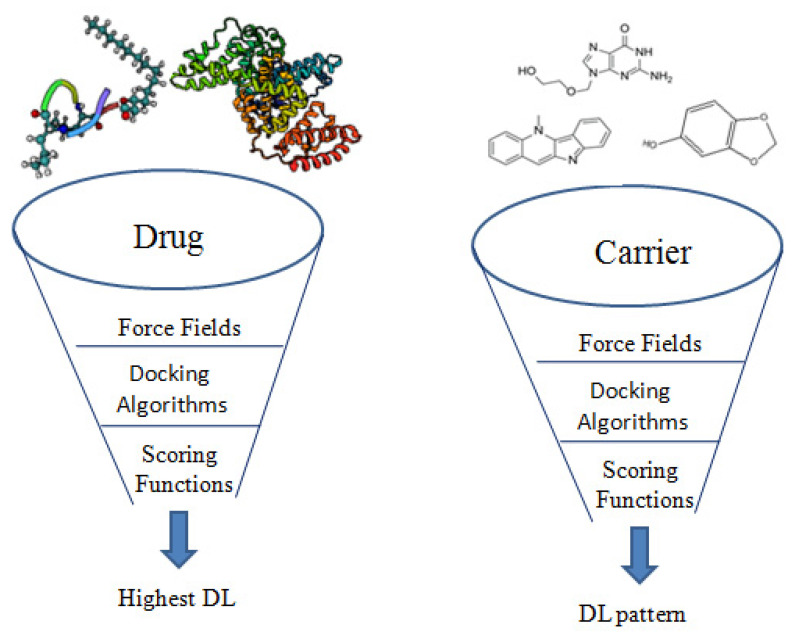
Screening role of different in silico parameters in the drug loading optimization of nanocarriers.

Different simulation protocols also strongly affect the success of modelling. A systematic study of twenty-one approved drugs was carried out by Sizochenko, and Leszczynski, [47] using again PLGA 50:50 as a carrier and AutoDock Vina for docking. A correlation of R^2^ = 0.36 (R = 0.6) was obtained between the literature gathered drug loading data and the resulted ∆G data. Compared to the correlation coefficient value in Metwally and Hathout report (0.9) [45] where the same carrier type and docking algorithm but different simulation force field were used- this recorded value was much lower. A force field (FF) is what defines the fundamental physics of the simulated system, i.e., determining the default values for physical interactions, bond lengths, partial charges and any force acting on/between atoms [48]. So, the quality of the FF will affect the accuracy and reliability of the simulated systems [28]. These approaches were summarized in Figure 3.

### 3.2. Visualizing Interactions

In addition to the screening and the carriers-selection tasks, simulations and docking are further used to gain insights into the mechanism by which binding processes occur. More specifically, the typical atomic/molecular level interactions between the drug and the carrier can be visualized and analyzed given the microscopic potential of these techniques [49]. From the following examples, it will be demonstrated how ΔG was related to different interactions in several studies, and how this relationship may contribute to the interpretation of unexpected loading cases of some compounds in different carriers.

In an important study, Brunacci et al. [50] conducted MD simulation to visualize possible interactions of dexamethasone in two polymers, PLGA 50:50 and oligobutylmorpholinediol (OBMD). They found that the amide group in OBMD acts as a hydrogen donor that supports the other hydrogen acceptor groups. Accordingly, a single OBMD unit was able to form three HB with a single dexamethasone molecule. In contrast, a PLGA unit that contains only hydrogen acceptors accompanied by its ester groups forms two HB with dexamethasone. Subsequent molecular docking analysis showed better ΔG of dexamethasone with OBMD compared to PLGA (Table 4). The experimental validation results, represented by EE and DL, ascertained the superiority of OBMD over PLGA to form dexamethasone-loaded polymeric nanoparticles (Table 4).

Also, Sonawane et al. [51] offered an excellent explanation of vancomycin loading pattern in compritol SLN, compritol-Eudragit RS 100 hybrid nanoparticles (LPHN) and compritol-PAMAM hybrid nanoparticles (LDHN). Simulations suggested that the only possible interaction between vancomycin and compritol was the weak hydrophobic interaction of vancomycin isopropyl moiety with compritol carbon chain backbone. In contrast, the highly branched PAMAM dendrimer was able to accommodate vancomycin molecules in its void spaces and back foldings. Analysis of the dendrimer-drug complex revealed that each dendrimer molecule was able to bind four vancomycin molecules by means of multiple HB (multivalent binding capacity). In the Eudragit core, vancomycin was not/barely able to form interactions despite the presence of active groups (alkyl groups and hydrogen acceptors (C=O)) in the Eudragit structure. In fact, the alkyl and hydrogen acceptor moieties of Eudragit were not practically available for interaction due to the steric hindrance effect (aligned chains/architecture of polymer with minimum void space c.f. dendrimer) especially with a bulky molecule like vancomycin. These findings were in line with the experimental results (Table 4).

Apart from physical interactions, other structural contributions like bulkiness, flexibility/rigidity and even isomerism of API can share in the final computation of ΔG. Costache et al.’s [37] previous perfect ranking (Table 3) was due to not only the binding interactions but also the drug bulkiness and flexibility. The size and flexibility of the three model drugs controlled their orientation and hence the inside tight binding in the nanocarrier core. Paclitaxel was theoretically able to form eighteen HB and many π-π interactions, but most of them were of intramolecular type due to its large size. This lead to a more rigid conformation of paclitaxel that mainly allowed of surface binding and therefore lower DL. The small rather flexible vitamin D3 then showed the best ΔG and DL (Table 4). Accordingly, it is evident that physical interactions solely are not sufficient to understand the binding affinity in nanosystems.

With more complex structural features, the affinity prediction job of binding energies becomes more difficult or even disabled, and counterintuitive experimental loading results can be observed. This can be clearly seen with Sánchez et al. [52] during the study of morphine and tramadol loading in PAMAM dendrimer. The experimental results showed a higher encapsulation proportion for morphine c.f. tramadol, while ΔG results exhibited more negative values for tramadol c.f. morphine (Table 5). This discrepancy was due to the possible drug-drug interactions of the racemic tramadol molecules. Based on this fact, an enantiomeric pair of tramadol can block the entranceway to the dendrimer cavity leading to the escape of molecules to the surrounding media despite the higher affinity of tramadol towards the dendrimer system as shown by ΔG values (Table 5).

A similar issue was encountered by Costache et al. [37] upon studying camptothecin docking on the PEG-tyrosine derived polyryltes-PEG carrier. The experimental results showed that camptothecin (small polycyclic and highly rigid drug) owned the lowest drug loading (Table 5) among the other three drugs (curcumin, paclitaxel and vitamin D3) (Table 4). Interestingly, camptothecin scored high ΔG (−9.27 kcal/mol) comparable to that of vitamin D3. Visualizing the atomic-level interactions revealed that for all docking replicates, camptothecin was preferably aligned in a specific spot of the carrier. About 70% of camptothecin hits accumulated in that “hot spot” with the remaining flew away the search space leading to false high scores. Translating this fact experimentally, nanoparticles would accommodate only few molecules of the drug and leak the rest to the surrounding media. The authors end with recommendations of searching and scoring refinement algorithms that consider not only the thermodynamic binding of molecules but also the kinetic view of binding [37].

Furthermore, melittin, a bee venom peptide, scored a higher ΔG upon docking on the simulated lecithin system c.f. a polymeric system (Table 5) [53]. However, the biophysical approach for drug loading measurement indicated lower drug loading in the case of lecithin c.f. the polymer. Upon visualizing molecular details of these interactions, the authors found that melittin interacts with the carriers by only certain residues critical for binding. Thus, there was an unnecessary overestimation of the contribution of bulkiness and flexibility of melittin molecules which lead to false better results with lecithin. These residues paralleled well with the polymer chain forming many interactions, but were loose with far intermolecular distances and only a few interactions in the case of lecithin. For further confirmation of these findings, the authors re-docked that specific residue of melittin sequence on lecithin and polymer systems. The obtained ΔG data flipped this time and matched the actual loading results (Table 5).

Dhanasekaran et al. [33] further went from modeling peptides to modeling the bulkiest, most complex pharmaceutical API, proteins. In their study, they applied MD simulation and docking calculations to predict insulin loading in both chitin and chitosan nanoparticles. They used HEX software for this purpose. Experimental loading results declared that chitosan was a better carrier for insulin c.f. chitin. Unfortunately, docking results failed to predict this finding. The obtained ΔG data was in contrast to the experimental loading results (Table 5). It seems that even the specialized protein docking servers were unable to account for the complexity of protein-polymer systems. A solution like that proposed by Misra et al. [53] may help.

Between success and failure stories of simulations and docking, the key strength and weaknesses at the same time of these techniques lie in their versatility. In docking, this can be best understood from the multiple engines available for conformations sampling (searching algorithms) and poses ranking (scoring functions) [54,55,56,57,58]. Similarly, diverse collection of force fields in the molecular simulation are also available besides different levels of resolution [28,46,59,60]. It is not so easy to predict the exact simulation-docking protocol suitable for a proposed situation, and many trials are sometimes needed for such a choice leading to high consumption of computational power and long timescales.

## 4. Artificial Intelligence and Machine Learning

**Figure 4:**
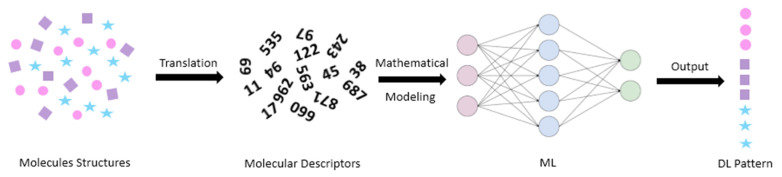
Molecular descriptors and machine learning in drug loading prediction.

Artificial intelligence (AI) is another field of problem solving used for complex multivariable data. The term originates from the fact it is a kind of simulation of human brain intelligence in machines that are programmed to think and act like humans. One great advance in this field is machine learning (ML), both supervised and unsupervised. Focusing on the first type, supervised ML is an area in AI which has the ability to learn and model the relationship between a set of independent variables and an outcome (response) [45]. The net result is a feature mapping equation that can predict outcomes for new observations [61,62,63,64]. In the world of drugs and carriers, supervised ML model inputs (independent variables) could be molecular descriptors and/or formulation parameters. Molecular descriptors are cheminformatics-based comprehensive numbers that abstract hundreds of structural characteristics of a compound [65]. It is ranging from simple bulk 1D features (molecular weight, polarizability, partition coefficient, etc) to the 2D features (number of atoms and bonds) and 3D features (pharmacophoric points and molecular fingerprints) [66].Generally, ML models are less computationally expensive compared to the combined MD and docking approach. In this context, ML techniques can provide results that are either complementary-to or comparable-with the MD and docking approach (Figure 4).

Hathout and Metwally experienced this concept in their previous work [45,46]. The authors represented the previously obtained ∆G by molecular descriptors of API and relate them using two supervised ML algorithms, an artificial neural network (ANN) algorithm [45] and a gaussian process (GP) algorithm [46]. Model inputs were the molecular weight, xlogP, topological polar surface area and fragment complexity of the literature gathered API, whereas model output was set as the obtained binding energies. This led to robust (Table 6) and fast estimation of ∆G without the need to encounter software limitations and high CPU computations cost during simulations and docking studies. Each descriptor was assessed for its significance to the differences in ΔG data. Subsequent loading prediction of API in the corresponding nanoparticles was encouraging (Table 3).

Also, Sizochenko, and Leszczynski [47] used molecular descriptors of their API as inputs in another supervised ML model against experimental DL as output. Briefly, they reflected API Van der Waals interaction potential by hydrophobicity (log P), miscibility and hydrogen bonds by lipophilicity (SIRMS-lip), electrostatic interactions by electronegativity (SIRMS-EO) and finally, they added a chirality representative of the studied compounds (Continuous Chirality Measure or CCM) as half of the used dataset were chiral drugs. They referred to the modeling algorithm as M5P. This algorithm combines a classification technique and a linear regression function at the nodes. This time, an excellent correlation between predicted and observed DL was obtained with R^2^ of >0.9 (Table 6). A Pearson evaluation of the effect of all descriptors on drug payload showed correlations of R > 0.9 indicating their great significance.

Actually, direct prediction of drug payload in nanoparticles from molecular descriptors of API is not a brand new idea. It was previously reported by Das et al. [67] on a dataset of twenty-two compounds using three different sets of molecular descriptors generated from three different software packages (Table 6). DRAGON descriptors were combination of 1D, 2D and 3D classes, while MOE generated 2D and 3D descriptors, and 2D descriptors only from VolSurf+. Each descriptors set was fitted in multiple linear regression model against the logarithmic form of the maximum attainable DL. After several permutations to exclude inter-correlations, each descriptors set was reduced to few non-correlated variables of primary significance on nanoparticles payload (Table 6). The success of the generated models was marked by R^2^ of >0.8 especially with the most inclusive set, DRAGON set (Table 6). This suggests that as more data from API becomes available, machine learning would become more powerful in the loading prediction job. Experimental validation was carried out using two drugs (silibin and andrographolide) and the observations correlated well with in silico predictive mass loading inside PLGA 50:50 nanoparticles.

Combining computed descriptors with experimental conditions, Cern et al. [68] developed two ML models to assess drug candidates that are able to achieve high remote (trans-membrane) loading in liposomes. A total of sixty drugs in three hundred sixty-six loading experiments were used to assemble a hybrid features model. Thus, molecular descriptors were combined with the experimental conditions for the entry in the dataset. Model output was set as the drug to lipid ratio (D/L) which is representative of the loading efficiency percentage. Determination coefficient (R^2^) and determination coefficient of the regression line forced to come through the origin (R^2^_0_) tested the predictive power of the model and retained a good fit between the predicted and observed target properties (Table 6).

Overall, these results are encouraging regarding payload prediction. Also, configuring the most important descriptors may be a mirror image to the possible interaction mechanism responsible for affinity during MD simulations and docking calculations [69,70,71,72,73,74].

## 5. Conclusions

In this review, an overview of the evolution of in silico formulation design technology in the investigation and prediction of drug payload in lipid and polymeric nanocarriers was presented. As illustrated, this technology is close to becoming routine add-ins for formulation scientists even for non-informatics specialists. They represent the next industrial revolution and deserve many other reviews to describe. Going from simple thermodynamic models to the 3D MD-docking approaches and AI-oriented algorithms, the extent to which a specific technique can guide the process of drug loading and formulation development have been explored. The researcher applying the idea has the welling to choose the most favorite/suitable technique from the above discussed ones or even merge between them.

## Figures and Tables

**Table 1:** Recent classical applications of solubility parameters and Flory-Huggins interaction theory for different lipid and polymeric nanocarriers and their main outcomes.

Intended Formulation	Carrier/Carriers	Applications	Drug/Drugs	Technique	Predictive Dry-Lab Values				Validating Wet-Lab Values		Correlation	Reference
PN LPHN	PLGA PLGA-Nigella sativa oil	Antioxidants, anti-inflamatory and anticancer	Indomethacin Curcumin Resveratrol α-tocopherol Hydrocortisone Retinol Izohidrafural Nitrofurantoin 5-fluorouracil	GCM based SP + FH interaction parameter	Δδ PN 0.51 0.37 2.68 1.84 4.04 1.77 0.76 12.14 15.17	Δδ LPHN 1.52 1.38 3.69 2.85 5.05 2.78 1.77 13.15 16.18	X_H_ PN 0.023 0.02 0.69 0.6 1.77 0.37 0.056 8.75 6.73	X_H_ LPHN 0.014 0.04 1.29 0.12 2.75 0.069 0.035 10.17 7.65	%EE * PN 35 25 20 25 28 79 42 16 5	%EE * LPHN 65 55 43 62 22 95 80 4 2	Exponential R^2^ 0.61 0.86 For PN and LPHN respectively	[17]
PM	PEG-PLA copolymer	Antifungals and anticancer	Diphenylpyrazole Pyrene Clotrimazole Plerixafor BLZ945 Combretastatin Niraparib Methoxytetralone Tranilast Plinabulin Curcumin Irinotecan Griseofulvin Cabazitaxel Heptylhydroxybenzoate Olaparib Paclitaxel Docetaxel Podophyllotoxin	GCM based SP + FH interaction parameter	Δδ 5.34 1.39 3.13 1.54 5 2.17 4.93 1.67 2 5.84 1.91 1.78 1.31 1.33 0.46 6.09 4.46 3.48 5		X_H_ 9.32 5.93 11.82 7.24 9.72 2.12 4.31 1.27 2.68 2.92 1.11 3.44 0.82 3.51 0.88 2.76 4.74 2.81 0.61		%DL 7.85 3.85 5.15 4.55 1.8 5.35 88.65 14.95 4.15 0.55 80.1 60.6 14.35 78.95 78.35 94.7 87.75 76.5 89.05		Prediction results did not align with the real experimental results.	[18]
PM	Poly-ethyloxazoline shell and butenyloxazoline, butyloxazoline, pentyloxazoline, or nonyloxazoline core	Anticancer	Curcumin	GCM based SP + FH interaction parameter	Δδ (core) 0.68 2.17 2.83 4.46 0.21		X_H_ (core) 0.05 0.48 0.82 2.03		%EE and %DL Upto 82.7% and 11.8% respectively for butenyloxazoline		-	[19]
SLN	Geleol Compritol ATO 888 Stearic acid Perciol Cetyl palmitate	Antibiotics, antivirals and corticosteroid	Minocycline- HCl Didanosine Efavirenz Clarithromycin Mometasone- furoate	Software-based Hansen SP (Yamamoto’s Molecular Breaking method in HSPiP software)	Δδ Geleol-mometasone 2.4 Geleol-minocyclin 8.65 Geleol-efavirenz 1.4 Geleol-didanosine 4.9 Compritol-didanosine 8 Stearic acid-clarithromycin 1.35				Quantity of lipid (gm) to solubilize 10 mg drug * 12 0.67 1.8 No solubility detected 3 3		No obvious prediction pattern was noticed. Bulky clarithromycin encountered some difficulties in Hansen calculations resulting in confusing predictions.	[20]

* Refers to estimated approximate values extrapolated from reference graphs or recalculated from the given data. PN: Polymeric nanoparticles; LPHN: Lipid-polymer hybrid nanoparticles; PM: Polymeric micelles; PLGA: Polylactic acid-co-glycolic acid; PEG-PLA: Polyethyleneglycol-polylactic acid; SLN: Solid lipid nanoparticles; SP: Solubility parameter; FH: Flory-Huggins; GCM: Group contribution method; Δδ: Difference in solubility parameters (drug and carrier); X_H_: Flory-Huggins interaction parameter; %EE: Entrapment efficiency percentage; %DL: Drug loading percentage.

**Table 2:** Simulations-derived solubility parameters and Flory-Huggins interaction theory for different nanocarriers and their outcomes.

Intended Formulation	Carrier/Carriers	Drug/Drugs	Technique	Predictive Dry-Lab Values		Validating Wet-Lab Values	Correlation	Reference
PM	PEO-b-PCL	Fenofibrate Nimodipine	MD simulation-based SP (COMPASS FF in MS software). GCM based SP and FH interaction parameter.	Δδ and X_H_ in MD 1.43–3.49 (0.05–0.33) 3–5.06 (0.23–0.68) respectively	Δδ and X_H_ in GCM 2.3 (0.539) 1.4 (0.25) respectively	Drug solubility in PCL core 120 mmol/mol 20 mmol/mol respectively	-	[29]
PM	PLA-PEG	Doxorubicin Cabazitaxel Beta-lapachone DrinabantSARRA	MD simulation-based Heldibrand SP and FH interaction parameter (COMPASS II FF in MS software)	X_H_ * 0.63 0.04 0.1 0.01 0.75 Near 0		%DL 1 5 2.2 7 2.7 10	Exponential correlation (no associated R^2^)	[30]
PN	PLLA PDLA PGA PEG Cellulose Chitosan	Cyclosporine A	MD simulation (PCFF or COMPASS FF in MS software) and docking-based (mixing energy in Blends module) SP and FH interaction parameter	X_H_ 103.6 169.2 219 206.9 17.7 43.7	ΔG −801.98 −749.86 −828.69 −723.35 −935.26 −957.61	Cited work	-	[31]

* Refers to estimated approximate values extrapolated from references’ graphs. PM: polymeric micelles; PN: polymeric nanoparticles; MD: Molecular dynamics; FH: Flory-Huggins; SP: Solubility parameter; GCM: Group contribution method; FF: Force field; MS: Materials studio software; Δδ: Difference in solubility parameters (drug and carrier); X_H_: Flory-Huggins interaction parameter; PLLA: Poly-L-lactic acid; PDLA Poly-D-lactic acid; PGA: Polyglycolic acid; PEG: Polyethyleneglycol; PEO-b-PCL: Polyethylene oxide-b-polycaprolactone copolymer; PLA-PEG: Polylactic acid-polyethylene glycol copolymer.

**Table 3:** Applications of molecular dynamic simulations coupled with docking techniques for modeling drug payload in different lipid and polymeric nanocarriers and their outcomes.

Intended Formulation	Carrier/Carriers	Drug/Drugs	Technique	Predictive Dry-Lab Values		Validating Wet-Lab Values	Correlation and Correlation Coefficient	Reference
PN PM	Chitosan Na alginate PLL NIPAAm-co-PEGPLGA	Curcumin	All-Atom MD simulation and docking (AutoDock Vina software)	ΔG (kcal/mol) −4.3 −3.3 −3.2 −2.9 −2.3		-	-	[32]
PN	Chitin Chitosan	Curcumin	MD simulation (MMFF in Schrodinger Macromodel software for pure components OPLS FF in Desmond software for carrier-drug complex) and docking (rigid docking in glide software)	ΔG (kcal/mol) −2.61 −3.31	HB count 3 6	%EE Up to 97.6% Up to 98.4%	-	[33]
PN	PLL PEA PAS PAA PEG-bis-amine PVA_2_ Chitosan Gelatin	Amphotericin B	Energy minimization of all built 2D structures (MMFF94 in ChemBioUltra software) and docking (AutoDock Vina software)	ΔG (kcal/mol) −3.1 −2.6 −3.4 −2.2 −1.7 −1.8 −3.3 −6.2		%EE and %DL in gelatin PN 78% and 2.42% respectively	-	[34]
PN	HPMC Eudragit EC PVA_1_ PVP Pluronics	Fluticasone	All-Atom MD simulation in Amber14 software package and docking (LGA) in AutoDock Vina software)	ΔG (kcal/mol) −35.22 for HPMC−25.17 for Eudragit		%EE >90% for both polymers	-	[35]
NanoMIPs	Poly itaconic acidPoly methacrylic acid Polyacrylamide	E-coli endotoxin	Energy minimization and docking screening (Tripos FF and LEAPFROG algorithm in SYBYL 7.0 software)	ΔG (kcal/mol) −52.24−41.43−39.87		Affinity by SPR 99.566.435.6	-	[36]
PM	PEG-Tyrosine derived polyarylates-PEG	Curcumin Paclitaxel Vitamin D3	All-Atom MD simulation (MMFF in MOE software) and docking (LGA in AutoDock 4 software)	ΔG (kcal/mol) −7.19 −4.36 −10.3		%DL * 29% 12% 36%	Linear R^2^ 0.93	[37]
PN	Chitin	Rifampicin Ethionamide Methacycline	MD simulation (CHARMM FF in discovery studio 4.0 software) and docking (AutoDock Vina software) and interaction visualization (PYMOL software)	ΔG (kcal/mol) −8.1 −7.3 −5.1		%DL 8.9% 5.6% 3.5%	Exponential/logarithmic R^2^ 0.85	[38]
PN	Chitin	Curcumin Docetaxel 5-fluorouracil	MD simulation (CHARMM FF in discovery studio 4.0 software) and docking (AutoDock Vina software) and interaction visualization (PYMOL software)	ΔG (kcal/mol) −6.9 −6.5 −5.4		%DL 3 2 1	Exponential/logarithmic R^2^ 0.93	[39]
PN	PLGA	Nine thiazoline derivatives	Hybrid QM/MM (ONIOM2 method in Gaussian 03 software using DFT and UFF)	ΔG (kcal/mol) D1: −8.1582 D2: −8.5694 D3: −9.0987 D4: −8.7034 D5 −8.0216 D6: −8.4022 D7: −9.4753 D8: −8.5970 D9: −8.2077		-	-	[40]
PM	PLA-PEG	Doxorubicin Cabazitaxel Beta-lapachone Drinabant SAR RA	MD simulation (Monte-Carlo method in MS software) and docking (metropolis Monte-Carlo surface docking in adsorption locator of MS software)	ΔG (kcal/mol) −29 −47 −40 From −109 to −115 −57 −100		%DL 1 5 2.2 10 2.7 7	Linear R^2^ 0.82	[30]
PN	Gelatin	Acyclovir Amphotericin B Cryptolepine Doxorubicin 5-fluorouracil Isoniazid Resveratrol Curcumin Paclitaxel Indomethacin	MD simulation (CGenFF in GROMACS software) and docking (AScore scoring function in ArgusLab software)	ΔG (kcal/mol) −3.94 144.4 −3.81 58.29 −4.19 −4.16 −3.74 −2.59 173.5 −1.99		DL (mg drug/100 mg gelatin) 8.74 1.16 2 2.1 25.07 22 1.96 3.5 0.52 1.91	Exponential R^2^ 0.95	[44]
SLN PN	Tripalmitin PLGA	Twenty-one literature gathered drug	All-Atom MD simulation (CGenFF in GROMACS software) and docking (ArgusLab and AutoDock Vina)	ΔG (kcal/mol)		DL Curcumin loading = 0.75 and 0.97 mg in 100 mg tripalmitin and PLGA respectively	Exponential R^2^ = 0.87 and 0.9 in SLN and PN respectively. % bias in loading = 12 and 2.03 in SLN and PN respectively	[45]
SLN	Tripalmitin	Ten literature gathered drugs	All-Atom MD simulation (CGenFF in GROMACS software) and docking (triangle matcher placement and ASE SF in MOE software)	ΔG (kcal/mol)		DL Curcumin loading = 0.81 mg in 100 mg tripalmitin	Exponential R^2^ 0.86 % bias in loading = 7.71	[46]
PN	PLGA	Twenty-one literature gathered drugs	MD simulation (UFF in Gaussview5 software) and docking (LGA in AutoDock Vina software)	ΔG (kcal/mol)		DL	Linear R^2^ 0.36 R 0.6	[47]

* Refers to estimated approximate values extrapolated from references’ graphs.MD: Molecular Dynamics; PN: Polymeric nanoparticles; PM: Polymeric micelles; SLN: Solid lipid nanoparticles; NanoMIPs: Molecularly imprinted polymer nanoparticles; %EE: Entrapment efficiency percentage; DL: Drug loading; HPMC: Hydroxypropylmethylcellulose; EC: Ethylcellulose; PAS: Polyaminostyrene; PAA: Polyallylamine; PEA: Polyethyleneamine; PLL: Poly-L-lysine; NIPAAM-co-PEG: N-isopropylacrylamide-co-polyethyleneglycol; PVA_1_:Polyvinyl alcohol; PEG: Polyethyleneglycol; PVA_2_: Polyvinyl amine; PVP: Polyvinylpyrrolidone; PLA-PEG: Polylactic acid-polyethylene glycol copolymer; PLGA: Polylactic acid-glycolic acid; MD: Molecular dynamics; FF: Forcefield; MS: Materials studio software; LGA: Lamarckian Genetic Algorithm; QM/MM: quantum mechanics/molecular mechanics; DFT: density functional theory; (%) DL: Drug loading (percentage) %EE: Entrapment efficiency percentage; HB: Hydrogen bond.

**Table 4:** Cases of binding site analysis in simulated lipid and polymeric nanocarriers and their observations.

Intended Formulation	Carrier/Carriers	Drug/Drugs	Technique	Predictive Dry-Lab Values (kcl/mol)	Validating Wet-Lab Values	Drug(s)-Carrier(S) Binding Interactions	Reference
PN	PLGA OBMD	Dexamethasone	MD (AMBER FF in Ascalaph designer software) and docking(AutoDock Vina) and interaction visualization (AutoDock visualizer tool)	ΔG −2.8 to −4.3 −3.8 to −5.1	%DL Up to 2 Up to 50	2 HB 3 HB	[50]
SLN LPHN LDHN	Compritol Compritol-Eudragit Compritol-PAMAM G4	Vancomycin	MD simulation (UFF in MS software) and docking (adsorption tool in MS software) and interaction visualization (Biovia discovery studio visualizer)	-	%DL 3.6 1.1 5.1	Hydrophobic interactions Nearly no interactions Multiple HB	[51]
PM	PEG-Tyrosine derived polyarylates-PEG	Curcumin Paclitaxel Vitamin D3	All-Atom MD simulation (MMFF in MOE software) and docking (LGA in AutoDock 4 software) and interaction visualization (AutoDock visualizer tool)	ΔG −7.19 −4.36 −10.3	%DL 29% 12% 36%	2 HB, 4 π-π interactions 1 HB, 2 π-π interactions 1 HB, 0 π-π interactions	[37]

LDHN: Lipid-dendrimer hybrid nanoparticles; PN: Polymeric nanoparticles; SLN: Solid lipid nanoparticles; LPHN: Lipid-polymer hybrid nanoparticles; OBMD: Oligobutylmorpholinediol; PEG: Polyethyleneglycol; PAMAM G4: 4th generation polyamidoamine dendrimer; %DL: Drug loading percentage; LGA: Lamarckian genetic algorithm; HB: Hydrogen bond; MD: Molecular dynamics; MS: Materials studio; FF: Force field.

**Table 5:** Cases of contradictory wet lab-dry lab results explained only by binding site analysis.

Intended Formulation	Carrier/Carriers	Drug/Drugs	Technique	Predictive Dry-Lab Values	Validating Wet-Lab Values	Reference
Dendrimers	PAMAM G5	Morphine Tramadol	MD simulation (CHARMM FF in NAMD software) and docking (LGA in AutoDock 4 software)	ΔG −0.97 to −11.79 −1.04 to −20.48	DL (moles drug/1mole dendrimer) 114 86	[52]
PM	PEG-Tyrosine derived polyarylates-PEG	Camptothecin	All-Atom MD simulation (MMFF in MOE software) and docking (LGA in AutoDock 4 software) and interaction visualization (AutoDock visualizer tool)	ΔG −9.27	%DL Up to 3%	[37]
Polybee nanoarchitecture Lipobee nanoarchitecture	PEG cetyl ether stabilized by either PS-PAA or lecithin	Melittin (bee venom peptide)	MD simulation (Tripos FF in SYBYL-X 2.0 software) and docking (induced fit, triangle matcher placement and London dG SF in MOE 2013 software)	1st ΔG +ve values −ve values 2nd ΔG −6.17 to −9.8−4.88 to −6.7	MTT assay (IC50) 40 and 80 nM 70 and 100 nM	[53]
PN	Chitin Chitosan	Insulin	MD (MMFF in Schrodinger Macromodel software for pure components and Optimized Potentials for Liquid Simulations (OPLS) FF in Desmond software for drug-carrier complex) and docking (SPF) and (FFT) algorithms in Hex software	ΔG −438.46 −420.69	%EE 80–83.97 86.4–89.13	[33]

PM: Polymeric micells; PN: Polymeric nanoparticles; PAMAM G5: 5th generation Polyamidoamine dendrimer; PEG: Polyethyleneglycol; PS-PAA: polystyrene-polyacrylic acid; LGA: Lamarckian genetic algorithm; %EE: Entrapment efficiency; (%) DL: Drug loading (percentage); MD: Molecular dynamics; FF: Force field; SPF: Spherical polar fourier; FFT: Fast fourier transform.

**Table 6:** Different applications of machine learning techniques for modelling lipid and polymeric nanocarriers.

Intended Formulation	Carrier/Carriers	Drug/Drugs	Technique	Predictive Dry-Lab Values	Validating Wet-Lab Values	Correlation	Reference
SLN PN	Tripalmitin PLGA	Twenty one literature gathered drug	Molecular descriptors calculation (Bioclipse software) and ANN model (jmp software)	Inputs Mwt, xLogP, TPSA and fragment complexity Output ΔG	% bias Up to 15% (3.66–14.9%)	R^2^ of the model: 0.999 0.999	[45]
SLN	Tripalmitin	Ten literature gathered drugs	Molecular descriptors calculation (Bioclipse software) and Gaussian process model (jmp software)	Inputs Mwt, xLogP, TPSA and fragment complexity Output ΔG	% bias 3.35%	Correlation between actual and predicted outputs: All the points were in close proximity to the 45^0^ line	[46]
PN	PLGA	Twenty one literature gathered drugs	Molecular descriptors and M5P QSPR model	Inputs logP, SiRMS-lip, SiRMS-EO and CCM Output DL	-	R^2^ between actual and predicted outputs >0.9	[47]
PN	PLGA 50:50	Twenty two literature gathered drug	Molecular descriptors (DRAGON, MOE and VolSurf+ programs) and MLR model (STATISTICA software)	Initial No. of descriptors 1504 201 128 Selected descriptors (inputs) Mor29u, GATS5m, C-019, T(N…O) and MATS2m E_Strain, Reactive, SMR_VSA4, MINDO_HF and SMR_VSA7 LgS10, WO6, DD4 and DRACAC Output Log DL		R^2^ of the model 0.889 0.826 0.818 for DRAGON, MOE anf VolSurf+ respectively	[67]
Liposomes	Different phospholipids	Sixty literature gathered drug	Molecular descriptors (MOE software) and kNN or SVR model (ChemBench software)	Inputs Hybrid of 185 1D-2D molecular descriptors, and eleven experimental conditions Output D/L mole ratio	-	R^2^ between actual and predicted outputs 0.758 0.789 R^2^_0_ between actual and predicted outputs 0.732 0.734 R^2^_0_ between actual and predicted outputs (without outliers) 0.919 0.883	[68]

PLGA: Polylactic acid-co-glycolic acid; SLN: Solid lipid nanoparticles; PN: Polymeric nanoparticles; ANN: Artificial neural network; MLR: Multiple linear regression; kNN: k-nearest neighbor; SVR: Support vector regression; QSPR: Quantitative structure-property relationship; Mwt: Molecular weight; TPSA: Topological polar surface area;Log P: Partition coefficient; CCM: Continuous chirality measure; DL: Drug loading.

## Data Availability

Not applicable.

## Abbreviations

(%)DL	drug loading (percentage)
(%)EE	entrapment efficiency (percentage)
AI	artificial intelligence
AlCIPc	Aluminium-chloride-phathalocyanine
Amber FF	assisted model building with energy refinement force field
ANN	artificial neural network
API	active pharmaceutical ingredient
CCM	continuous chirality measure
CD	cyclodextrin
CED	cohesive energy density
CGen FF	CHARMM general force field
CHARMM FF	chemistry at Harvard macromolecular mechanics force field
DDS	drug delivery system
DFT	density function theory
EC	ethylcellulose
Emix	mixing energy
FF	force field
FFT	fast fourier transform algorithm
FH	Flory-Huggins theory
GCM	group contribution method
GP	Gaussian process
HB	hydrogen bond
HPMC	hydroxypropylmethyl cellulose
HSPiP	Hansen solubility parameter in practice
KNN	k-nearest neighbor
LDHN	lipid-dendrimer hybrid nanoparticles
LGA	Lamarckian genetic algorithm
LPHN	lipid-polymer hybrid nanoparticles
MD	molecular dynamics
ML	machine learning
MLR	multiple linear regression
MMFF	Merck molecular force field
MOE	molecular operating environment
MS	materials studio
Mwt	molecular weight
NanoMIPs	molecularly imprinted polymeric nanopartices
NIPAAm	N-isopropylacrylamide
OBMD	oligobutylmorpholinediol
ONIOM	our N-layered integrated molecular orbital+molecular mechanics
OPLS FF	optimized potentials for liquid simulations force field
PAA	Polyallylamine
PAMAM	polyamidoamine
PAS	Polyaminostyrene
PCFF	polymer consistent force filed
PCL	polycaprolactone
PDLA	poly-D-lactic acid
PEA	Polyethyleneamine
PEG	polyethylene glycol
PEO	polyethylene oxide
PGA	polyglycolic acid
PLGA	polylactic-co-glycolic acid
PLL	poly-L-lysine
PLLA	poly-L-lactic acid
PM	polymeric micelles
PN	polymeric nanoparticles
PS-PAA	polystyrene-polyacrylic acid
PVA1	polyvinyl alcohol
PVA2	polyvinyl amine
QM/MM	quantum mechanics/molecular mechanics
QSPR	quantitative structure-property relationship
R/R2	correlation/determination coefficients
SF	scoring function
SLN	solid lipid nanoparticles
SP/δ	solubility parameter
SPF	spherical polar fourier algorithm
SPR	surface plasmon resonance
SVR	support vector regression
TPSA	topological polar surface area
UFF	universal force field
XH	Flory-Huggins interaction parameter
xLog P	partition coefficient
Δδ	difference in solubility parameters (drug and carrier)
ΔG	binding energy
